# Resistance Against *Leishmania major* Infection Depends on Microbiota-Guided Macrophage Activation

**DOI:** 10.3389/fimmu.2021.730437

**Published:** 2021-10-20

**Authors:** Mateus Eustáquio Lopes, Liliane Martins dos Santos, David Sacks, Leda Quercia Vieira, Matheus B. Carneiro

**Affiliations:** ^1^ Laboratório de Gnotobiologia e Imunologia, Instituto de Ciências Biológicas, Departamento de Bioquímica e Imunologia, Universidade Federal de Minas Gerais, Belo Horizonte, Brazil; ^2^ Laboratory of Parasitic Diseases, National Institute of Allergy and Infectious Diseases, National Institutes of Health, Bethesda, MD, United States

**Keywords:** *Leishmania major*, macrophage, monocyte, cell-activation, nitric oxide, microbiota

## Abstract

Innate immune cells present a dual role during leishmaniasis: they constitute the first line of host defense but are also the main host cells for the parasite. Response against the infection that results in the control of parasite growth and lesion healing depends on activation of macrophages into a classical activated phenotype. We report an essential role for the microbiota in driving macrophage and monocyte-derived macrophage activation towards a resistance phenotype against *Leishmania major* infection in mice. Both germ-free and dysbiotic mice showed a higher number of myeloid innate cells in lesions and increased number of infected cells, mainly dermal resident and inflammatory macrophages. Despite developing a Th1 immune response characterized by the same levels of IFN-γ production as the conventional mice, germ-free mice presented reduced numbers of iNOS^+^ macrophages at the peak of infection. Absence or disturbance of host microbiota impaired the capacity of bone marrow-derived macrophage to be activated for *Leishmania* killing *in vitro*, even when stimulated by Th1 cytokines. These cells presented reduced expression of *inos* mRNA, and diminished production of microbicidal molecules, such as ROS, while presenting a permissive activation status, characterized by increased expression of *arginase I* and *il-10* mRNA and higher arginase activity. Colonization of germ-free mice with complete microbiota from conventional mice rescued their ability to control the infection. This study demonstrates the essential role of host microbiota on innate immune response against *L. major* infection, driving host macrophages to a resistance phenotype.

## Introduction

Monocytes and macrophages are mononuclear phagocytic cells that play an essential role during both homeostasis and during inflammation by promoting pathogen killing and participating in tissue regeneration (1, 2). These cells present plasticity in activation phenotypes depending on the environmental stimuli. When activated by IFN-γ and TNF or Toll-like receptors (TLR) ligands these cells respond by expressing iNOS, leading to the metabolism of l-arginine towards nitric oxide production. They also show increased production of reactive oxygen species (ROS), as well as higher levels of TNF, IL-12 and chemokines such as CXCL9 and CXCL10, characterizing a pro-inflammatory phenotype (3). On the other hand, when activated by cytokines such as IL-4 and IL-13 the macrophages acquire a wound-healing function, upregulating genes such as *retnlα* (*fizz1*), *pdl2*, *socs2*, *chil3* and *ccl24*. In addition, these cells express higher levels of arginase I (Arg1), which metabolizes l-arginine into polyamine production. Macrophages can also get activated by TLR ligands in the presence of immunocomplexes, acquiring a suppressive phenotype characterized by production of high levels of IL-10 (2–5). The activation status of macrophages during infectious diseases plays a major role in either promoting parasite killing or replication, especially in the context of phagosomal pathogens, such as *Leishmania*.

Leishmaniasis is a vector-borne disease caused by protozoan parasites of the genus *Leishmania*. Depending mostly on the parasite species, clinical manifestations of the disease can range from cutaneous, muco-cutaneous and visceral forms (6). *Leishmania* are phagosomal pathogens that are adapted to survive and replicate inside the hostile environment of mature phagolysosomes. Different phagocytic cells can uptake *Leishmania* through the course of infection, including neutrophils, tissue resident macrophages (TRMs), monocytes, monocyte-derived cells and dendritic cells (7, 8). Despite the innate effector mechanisms associated with phagocytosis, such as ROS production and the formation of a mature phagolysosome, most parasites survive (7, 9, 10) and the development of an adaptive immune response is required for the elimination of parasites (11). During chronic stages of the disease by different species of *Leishmania*, monocytes and macrophages, either embryonic-derived or monocyte-derived, represent critical host cells for the parasite, as they can become host cells for parasite replication or get properly activated and mediate parasite killing (12–17). In this context, the development of a Th1 immunity is essential for resistance against *Leishmania* (18–20). The activation of host phagocytic cells by IFN-γ produced by Th1 cells inducing NO production can either directly damage the parasites or restrict their growth by impacting their metabolism (21). On the other hand, activation of Th2 immunity is associated with a non-protective phenotype during *Leishmania* infection. Th2 derived cytokines, such as IL-4, can induce arginase I activation, which can favor parasite growth (22, 23). The Th1/Th2 dichotomy, however, does not always explain infection outcome in mice. For example, even during a highly polarized Th1 immune response, the Seidman strain of *L. major* can cause non-healing cutaneous lesions by preferentially infecting TRMs that maintain a wound healing phenotype despite the Th1 environment in which they are found (24). In addition, our group has shown that in the absence of the microbiota the Th1 immune response is not sufficient to control the disease in Swiss mice (25).

Host microbiota exerts a vast influence on the immune system, starting before birth and maintained through organism development (26–28). For instance, during steady state, myeloid cells generation is influenced by the systemic presence of microbiota compounds and metabolites (29–32). Hematopoietic stem cells and embryo-derived myeloid cells are sustained at steady state by commensal microbiota (30, 32). Granulopoiesis is driven by microbial compounds present in the serum through activation of TLR and MyD88-TICAM1 signaling pathways (29). Microbial compounds also drive the emigration of monocytes from bone marrow and influence neutrophils, monocytes and macrophages within systemic sites (30, 31). Those compounds drive the inflammatory responses promoting antibody and cytokine production (28). Constitutive antibody against host microbiota that acts controlling the microbiota pool also presents a role during inflammation by blocking gut microbe translocation and preventing infections against exogenous pathogenic bacteria *via* recognition of conserved molecules in the microbe membrane (33). Commensal-derived peptidoglycans present in the serum improve neutrophil-killing ability against *Streptococcus pneumonia* and *Staphylococcus aureus* infection through cell activation by LPS and LTA (34). Moreover, monoassociation of germ-free mice with *Lactobacillus delbrueckii* improve the immune response against *Lysteria monocytogenes* infection (35). There are few studies addressing the role of microbiota in leishmaniasis and disease outcome (36). Oliveira et al. (25) showed that the microbiota plays an important role in a self-healing experimental *L. major* infection in mice independent of Th1 response in the well-described footpad infection model. Using the intradermal route of infection, Naik et al. (37) showed that in germ-free C57BL/6 mice host cells are permissive to parasite replication even though they develop smaller lesions and less necrosis than conventional mice. Monoassociation of germ-free mice with *Staphylococcus epidermidis* on the skin recovers their ability to control the parasite, and the local skin microbiota seems to be more important in leishmaniasis than the gut microbiota in *Leishmania major* infection (37). In humans, cutaneous leishmaniasis lesions shape the local microbial composition which can extend to adjacent areas of healthy skin. The dysbiosis is marked by increased specific groups of bacteria that were related to the outcome of disease (38). Gut microbiota composition seems to be relevant in visceral leishmaniasis since it was recently shown that gut microbiota modulation by an antibiotic cocktail can improve the disease outcome in hamsters infected with *L. donovani* (39).

As macrophages are the main host cell for *Leishmania sp*, it is possible that modulation of macrophage function by the host microbiota might contribute to the effects that the microbiota has on infection outcome. Macrophages from germ-free mice show increased wound-healing phenotype and can be associated with greater resolution of skin inflammation after excisional injury (40). In addition, peritoneal-derived macrophages from germ-free mice presented reduced *in vitro* killing of *L. major* after IFN-γ stimuli (25) and impaired ability to eliminate other intracellular pathogens after pro-inflammatory stimuli (41, 42). We hypothesize that the host microbiota might play a role in driving macrophage activation towards a resistant phenotype in the context of Th1 immunity, which would explain why germ-free mice are susceptible despite the production of equal levels of IFN-γ, IL-12 and TNF compared to conventional mice (25). Using different experimental strategies, including germ-free mice, the treatment of conventional mice with antibiotics, or colonization of germ-free mice after weaning by fecal transfer, this work sought to elucidate how the microbiota influences macrophage function during *L. major* infection, and how this impacts on the development of disease.

## Material and Methods

### Mice

Swiss/NIH germ-free, female mice were acquired from Taconic (Madison, WI, USA) and kept sterile using gnotobiology techniques (43). Germ-free and conventional Swiss/NIH females, 6-8 weeks old, were used in all experiments. BALB/c female mice 6-8 weeks old were used to maintain the parasite infectivity and acquired from Centro de Bioterismo, Instituto de Ciências Biológicas (CEBIO), UFMG. Mice were kept in the animal facility from Departamento de Bioquímica e Imunologia, UFMG with sterile water and food (Nuvilab, Curitiba, PR, BR) *ad libitum*. All procedures were approved by CEUA/UFMG 110/2012 and 266/2017 protocols.

### Host Microbiota Modulation

Microbiota depletion in conventional Swiss/NIH female mice was performed using antibiotic cocktail administration in sterile water. The cocktail was composed of metronidazole (1g/L; Sigma-Aldrich, St. Louis, MO, USA), neomycin (1g/L; Sigma), ampicillin (1g/L; Sigma) and vancomycin (0.5g/L; Sigma). Conventional and antibiotic-treated groups received sucralose (1.5g/L; Linea, Embu das Artes, SP, BR) in water. Treatment started after weaning and was continued until mice were 6 weeks old, when they were infected. Antibiotic treatment continued up to 6 weeks post-infection. For bone marrow-derived macrophage assays, 6-8 weeks old antibiotic treated mice were used. To confirm microbiota depletion, fecal samples were collected throughout the depletion process and on the day of the experimental procedures and cultured using MacConkey (HiMedia, Kennett Square, PA, USA), brain heart infusion (HiMedia) and *Lactobacillus* MRS (BD, Sparks, MD, USA) agars (**Supplementary Figure 3A**).

Fecal microbiota transfer was performed to reconstitute germ-free microbiota. Feces from germ-free 4 week old conventional mice were collected in the hood. Then, inside the anaerobic chamber (Thermo Scientific, Wilmington, DE, USA) feces were resuspended in PBS, homogenized, and filtered in a 40µm cell strainer to remove larger particles. Germ-free mice at 4 week old received 100µL of this solution by oral gavage, twice at one week interval. Mice that received the microbiota transfer were kept in the same cages as the microbiota donors for 2 weeks, to improve the reconstitution. Mice were then separated and infected as described below.

### *Leishmania major* Culture, Infection, Antigen Generation

*Leishmania major* MHOM/80/Friedlin wild type or transfected with plasmid containing Red Fluorescent Protein (RFP) were defrosted and cultured in 199 culture media (GIBCO, Grand Island, NY, USA) supplemented with 10% FBS (Cultilab, Campinas, SP, BR), 2mM of l-glutamine (GIBCO), 100U/L of penicillin (GIBCO), 100µg/mL of streptomycin (GIBCO), 1% of 10mM adenine (Sigma), 0.2% of hemin (Sigma), 0.25% of 10mM NaOH, 0.1% of biotin (Sigma). For the RFP *L. major*, 50 µg/mL of geneticin (GIBCO) were added to the medium. After 7 days in culture, the promastigotes were inoculated into BALB/c mice footpads to maintain the parasite infectivity. 4 to 5 weeks post infection the BALB/c mice were anesthetized by intraperitoneal injection of 100µL Ketamine (15mg/mL; Vertbrands, Miramar, FL, USA) and Xylazine (5mg/mL; Vertbrands) solution. Footpads were aspirated by syringe to collect the parasites that were grown on culture medium until the promastigote stage and transferred to tissue culture flasks. Promastigotes were kept at 25°C and transferred to new medium every 3 days. To perform infection, parasites were kept for 5 days in culture, and the metacyclic promastigotes were obtained as previously described (44). Mice were infected with 10^6^ metacyclic forms into the footpad and the lesion was measured with a caliper every following week until 10 weeks after infection. Every two weeks a group of mice was sacrificed, the footpads were harvested for parasite quantification by limiting dilution. A two-fold serial dilution was performed in 96 well plates with 199 culture media. Parasite growth was observed at 7 and 10 days after incubation, the number of viable parasites was determined by the highest dilution at which parasite growth was observed and expressed by the mean values of negative log_10_ of the titer (45).

Parasite antigens were obtained from promastigote cultures at day 5 using centrifugations and 7 freeze-thaw cycles were performed. Protein amounts were measured using the Lowry method and aliquots with 1mg/mL were stored at -20°C until use (20).

### Popliteal Draining Lymph Node Cell Culture

Popliteal draining lymph nodes were harvested from naïve and infected mice every 2 weeks following infection. After processing and cell isolation, 5x10^6^ cells/mL per well were plated in RPMI 1640 medium (GIBCO) supplemented with 10% FBS (Cultilab), 2mM of l-glutamine (GIBCO), 100U/Lpenicillin (GIBCO), 100µg/mL streptomycin (GIBCO) and 25mM HEPES (Sigma). The culture was stimulated with 50µg/mL *L. major* (L.m) or *L. major* RFP (L.m RFP) antigens, then the supernatant was collected 24h and 72h after stimulation for cytokine measurement by ELISA.

### Flow Cytometry

Naïve and infected footpads were harvested every 2 weeks after infection with *L. major* or *L. major* RFP. Samples were digested with 62.5µg/mL liberase TL (Roche, Mannheim, GE) and 0.5mg/mL of DNAse I (Sigma) in RPMI 1640 (GIBCO) for 1h30min at 37°C. After digestion, the enzymatic reaction was terminated by adding RPMI 1640 containing 5% of FBS (Cultilab). The samples were homogenized and centrifuged for 10 minutes at 400xg at 4°C. The pellet was washed, cells were counted, and 10^6^ cells were plated in 96-well plate (Corning, New York, NY, USA). Surface staining was performed for 20min at 4°C using Fc block Fc-γ III/II CD16/32 (clone 2.4G2; BD), anti-Ly6G (clone 1A8; BD); anti-Ly6C (clone HK1.4; BD); anti-F4/80 (clone BM8; eBioscience, San Diego, CA, USA); anti-CD11c (clone HL3; BD); anti-CD11b (clone M1/70; BD); anti-CD8 (clone 53-6.72; BD); anti-CD3 (clone 145-2C11; BD); anti-CD19 (1D3; BD); anti-CD4 (clone GK1.5; BD). For intracellular staining samples were fixed and permeabilized using Fix/Perm Kit from BD, then stained with anti-NOS2 (clone CXNFT; eBioscience). Samples were acquired using FACs Canto II (BD).

### Culture and Activation of Bone Marrow-Derived Macrophages

Bone marrow precursors were harvested from femurs, washed and differentiated to macrophages in culture dish containing DMEM-F12 (GIBCO) supplemented with 10% FBS (Cultilab), 2mM of l-glutamine (GIBCO), 100U/L penicillin (GIBCO), 100µg/mL streptomycin (GIBCO), 25mM HEPES (Sigma), and 20% of supernatant from L929 cell culture as previously described (46). After nine days of differentiation at 37°C, 5% CO_2_ incubator, cells were harvested and stimulated with 50U/mL IFN-γ (BD) plus 100ng/mL of Lipopolysaccharide (LPS; InvivoGen, San Diego, CA, USA); 40U/mL of IL-4 (BD); or 0.05mg/mL prostaglandin E2 (PGE_2_; Cayman, Ann Arbor, MI, USA) plus 100ng/mL of LPS.

### ELISA

Measurement of cytokines was performed in the bone marrow-derived macrophages (BMDM) and popliteal draining lymph node cell culture supernatants. For TNF, supernatant was collected 24h after culture, the other cytokines were measured on 48h supernatants, performed according to manufacturer instructions (BD). The limit of detection was 32pg/mL for all cytokines.

### Biochemical Assays

Nitric oxide was indirectly measured by nitrites quantification using Griess reaction as previously described (47). Detection was performed on bone marrow derived-macrophage culture supernatant 48 hours after stimulation. Standard curve was generated by serial dilution of sodium nitrite from 250µM to 1,95µM. Measurement was performed at 540nm in a spectrophotometer.

Reactive oxygen species (ROS) were measured on BMDM stimulated or not with IFN-γ + LPS. Zymosan particles stimuli, 10^7^ particles/in 50µL, were used as positive control for ROS production. Measurements were performed using Luminol technique (48). Briefly, cells were incubated in 96 well opaque plate (Corning) with RPMI without phenol red (GIBCO) and the stimuli, then 0.05mM luminol (Sigma) was added in each well. ROS production was measured by light emission detected by the Luminometer (Packard, Waltham, MA, USA) with 2 min interval between each record for 2 hours and represented as Relative Light Units (RLU).

Arginase activity was performed on processed footpad samples from naïve and infected mice at each time point post-infection and BMDM cell culture homogenates 48 hours after IL-4 stimuli. The protocol was previously described (49) with minimum modifications (45). Results were expressed as µmols of urea produced per minute. Standard curve was generated by serial dilution of urea and the limit of detection was 270 µL. Measurement was performed at 540nm in a spectrophotometer.

### Analysis of mRNA Expression by Real Time PCR

Total mRNA was extracted from BMDM 24h after the different stimuli using Trizol (Invitrogen, CarlsBad, CA, USA) extraction (20). Using RT-PCR, the cDNA was generated, amplified using SYBER Green (Applied Biosystems, Foster City, CA, USA) during 45 cycles of 2 minutes at 50°C, 10 minutes at 95°C, 1 minute at 60°C, and further amplified by a cycle of 15 seconds at 95°C followed by 5 seconds at 60°C and finally 1 minute at 4°C. The amplifications were performed at Q-PCR 7500 Applied Biosystems Instrument. The results were analyzed by the comparative threshold cycle method using 2^-ΔΔCT^ to determine the fold increase (20). Each gene expression was normalized to *act b* mRNA expression as endogenous control and non-stimulated cells. Evaluated genes and their sequences are described in **Supplementary Table 1**.

### Statistical Analysis

Data were analyzed using GraphPad Prism 7.0 (GraphPad Software, San Diego CA, USA) and flow cytometry data was analyzed using FlowJo v10 (Tree Star Inc, Ashland, OR, USA). All data were submitted for normality testing, the parametric data was shown as SEM and non-parametric as Median. In two group comparisons, t-student or Mann-Whitney tests were performed. For kinetic and multiple groups assays, we performed Two-Way Anova followed by Bonferroni post-test in parametric data and Kruskal-Wallis for non-parametric data. Differences were considered statistically significant when p<0.05 (*); p<0.01 (**); p<0.001 (***); p<0.0001 (****).

## Results

### Resistance to *Leishmania major* Infection Depends on the Host Microbiota

**Figure 1:**
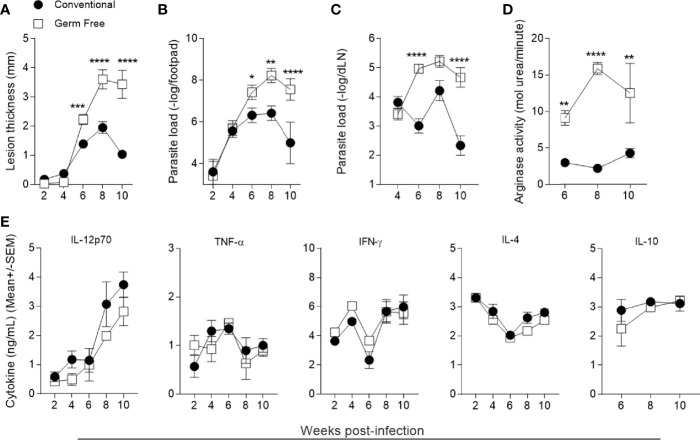
Germ-free mice polarize immune response to Th1 but are still susceptible to *L. major* infection. **(A)** Lesion thickness in footpads was monitored every two weeks up to 10 weeks post infection. **(B)** Quantification of parasite loads in footpads by limiting dilution analysis. **(C)** Quantification of parasite loads in popliteal draining lymph nodes by limiting dilution analysis. **(D)** Arginase activity in footpads 6, 8 and 10 weeks after infection. **(E)** Cytokine production by popliteal lymph node cell culture supernatants 2, 4, 6, 8 and 10 weeks after infection 72h (IFN-γ, IL-12p70, IL-4 and IL-10) and 24h (TNF) after stimulation with *L. major* antigens. *, **, ***, **** indicates statistical difference between the two groups at the same time point using two-way ANOVA and post-Bonferroni test (p < 0.05; p < 0.01; p < 0.001; p < 0.0001). Results are representative of three independent experiments. Three to5 animals were used per group in each time point.

As previously observed, in the absence of the microbiota germ-free (GF) mice are susceptible to *L. major* infection (25). After infection with 10^6^ metacyclic promastigotes of *L. major*, GF mice developed larger lesions from the sixth to the tenth week (**Figure 1A**). Quantification of parasite load followed the same profile as the course of lesion development: GF mice presented higher number of parasites in the footpad and popliteal lymph node after six weeks post-infection (**Figures 1B, C**). The increase in parasite burden was associated with increased arginase I activity in the lesions (**Figure 1D**), indicating a greater availability of polyamines which are used by intracellular parasites to multiply (23, 49, 50).

**Figure 2:**
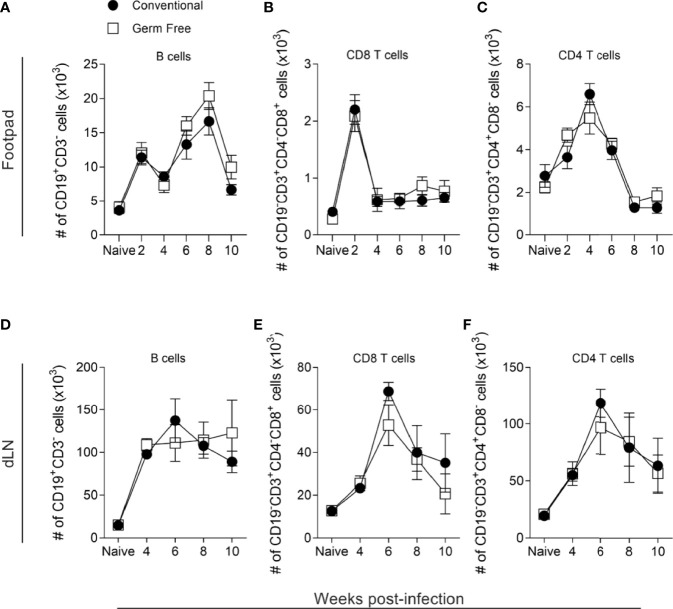
Host microbiota did not alter lymphocyte infiltrates in lesions or draining lymph nodes. **(A–C)** Absolute number (#) of lymphocytes in the footpads of uninfected and infected mice, 2-10 weeks after infection. **(D–F)** Absolute number (#) of lymphocytes in the popliteal lymph nodes (dLN) of uninfected and infected mice, 4-10 weeks after infection. Groups of mice were compared at the same time point using two-way ANOVA and Bonferroni post-test (p < 0.05). Results are representative of two independent experiments. Four to 6 animals per group were used per time.

The establishment of a predominant Th1 environment at the infection site is associated with resistance against *L. major* infection (51, 52). To address if the higher susceptibility of GF mice was due to an impaired Th1 immune response, supernatants of lymph node cell cultures stimulated *in vitro* with *L. major* antigens were analyzed for production of cytokines. GF mice produced similar levels of both pro- and anti-inflammatory cytokines (**Figure 1E**) compared to conventional (CV) mice throughout the course of infection. Specifically, GF animals produced a comparable Th1 (IL-12p70, IFN-γ) and inflammatory cytokine (TNF) response as CV animals, suggesting that the mechanism of susceptibility conferred by the GF status is not due to lack of development of a Th1 immune response. In addition, no differences were found regarding IL-4 and IL-10 production between the groups (**Figure 1E**). Analysis of lymphocyte populations within lesion and in popliteal lymph node (**Supplementary Figure 1A**) showed that GF mice had similar numbers and frequencies of B, CD8+ T, CD4+ T and regulatory T lymphocytes (**Figures 2A–F** and **Supplementary Figures 1B–G**) along the course of infection. Taken together, these data suggest that the microbiota did not influence the development of the adaptative immune response against *L. major*.

### Phagocytic Cells Are More Permissive to *Leishmania major* Infection in the Absence of Host Microbiota

**Figure 3:**
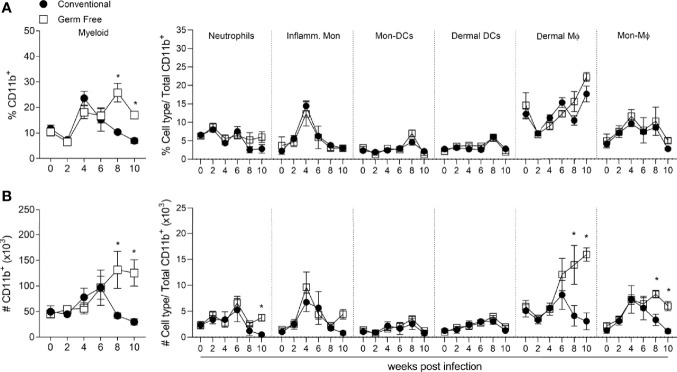
Germ-free mice showed higher number of myeloid cells at late infection time points. **(A)** Percentage of myeloid cells subsets in the footpad of uninfected and *L. major* infected mice, 2-10 weeks after infection. **(B)** Absolute number (#) of myeloid cells subsets in the footpad of uninfected and *L. major* infected mice, 2-10 weeks after infection. * indicates statistical difference between groups of mice using two-way ANOVA and Bonferroni post-test (p < 0.05). Results are representative of two independent experiments. Four to 6 animals per group were used per time.

Due to the similarity between GF and CV adaptive immune response to *L. major*, we decided to characterize innate immune cell populations present in the lesions during the infection. Neutrophils (7), monocytes (13), macrophages (8, 14), and dendritic cells (53) are components of the myeloid immune system and play important roles in the innate immune response against intracellular pathogens, including *L. major* (54, 55). Kinetics of these cells during infection was addressed according to the gating strategy shown in **Supplementary Figure 2A**. GF mice presented higher proportion (**Figure 3A**, left panel) and greater numbers (**Figure 3B**, left panel) of CD11b^+^ myeloid cells at late stages of the disease, which can be associated with larger lesions observed in GF mice (**Figure 1A**) 8 and 10 weeks post-infection (**Figures 3A, B**). The frequency of the different myeloid cell populations did not differ between the groups at any time points (**Figure 3A**, right panel). However, in accordance with the higher number of CD11b^+^ cells in GF mice, we found higher numbers of neutrophils, dermal macrophages (dermal Mϕ) and monocyte-derived macrophages (Mon-Mϕ) at later times of the infection (**Figure 3B**, right panel). Neutrophil infiltrate was not different for most of the course of infection, except for the tenth week. After 8 weeks post-infection, we observed a sustained greater number of both dermal macrophages and monocyte-derived macrophages (**Figure 3B**) in the lesions of GF compared to CV mice. Similar numbers were observed in the dermal dendritic cells and monocyte-derived dendritic cell populations. The higher numbers of neutrophils, dermal Mϕ and Mon-Mϕ might explain the persistence of larger lesions and parasites in GF mice.

**Figure 4:**
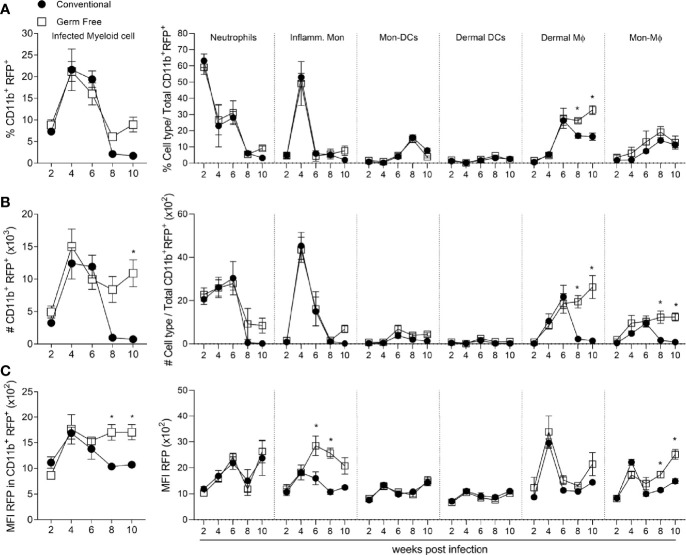
Germ-free monocytes and macrophages remain infected after peak of infection. **(A)** Percentage of infected myeloid cells subsets in the footpad of infected mice 6-10 weeks after infection. **(B)** Absolute number (#) of infected myeloid cells and their subsets in the footpad of infected mice 2-10 weeks after infection. **(C)** MFI of RFP in infected myeloid cells and each immune cell subset in the footpad of infected mice 2-10 weeks after infection. * indicates statistical difference between groups of mice using two-way ANOVA and Bonferroni post-test (p < 0.05). Results are representative of two independent experiments. Four to 6 animals per group were used per time.

To evaluate the relevance of each cell type as a potential niche for parasite replication during infection, a *L. major* strain that expresses a plasmid encoding red fluorescent protein (Lm RFP^+^) was used (7), thus enabling the evaluation of infected cells by flow cytometry (gating strategy in **Supplementary Figure 2B**). By gating on CD11b^+^RFP^+^ cells we could then discriminate the different cell types of myeloid cells which were infected (**Figures 4A, B**). Importantly, the frequency and numbers of infected cells in CV mice dropped dramatically after 8 weeks post-infection (**Figures 4A, B**), which correlates with the reduction in lesion size (**Figure 1A**) and total parasite load (**Figure 1B**). By contrast, the GF mice did not show a decrease in numbers of infected cells from 6 to 10 weeks post-infection, which correlates with their susceptible phenotype (**Figures 1A, B**). Thus, in later time points of infection (after 8 weeks), a higher frequency and number of infected cells were observed in GF compared to CV mice (**Figures 4A, B**). By identifying the different RFP^+^ myeloid cell subsets we did not observe differences in either the frequency or numbers of infected neutrophils, inflammatory monocytes, dermal DCs or Mon-DCs (**Figures 4A, B**). GF mice did, however, have a greater proportion and higher numbers of infected dermal Mϕ at 8- and 10-weeks post-infection (**Figures 4A, B**). In addition, GF mice showed higher numbers of infected Mon-Mϕ after 8 weeks post-infection (**Figure 4B**). Lastly, after 8 weeks post-infection in the GF mice, the median fluorescence intensity (MFI) of RFP expression was increased in the total myeloid population, primarily within the Mon-Mϕ (**Figure 4C**). Taken together, our data suggest that inflammatory monocytes and both dermal and monocyte-derived macrophages are more permissive to *L. major* in the absence of microbiota, even in the context of a Th1 immune response, and represent an important niche for parasite persistence at late time points of infection.

**Figure 5:**
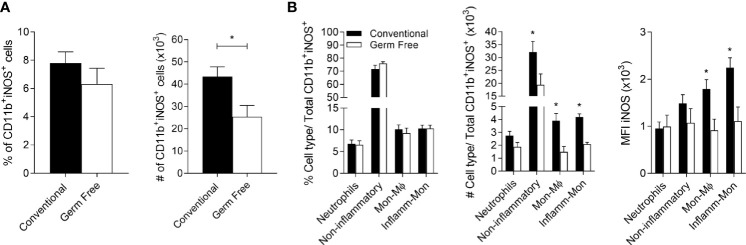
Germ-free mice present fewer microbicidal monocytes/macrophages. **(A)** Percentage (%) and absolute number (#) of CD11b ^+^iNOS^+^ cells in the footpads of mice six weeks after infection with *L. major* Wt. **(B)** Percentage (%) and absolute number (#) of CD11b^+^iNOS^+^ cells in the footpads of mice 6 weeks after infection. MFI of iNOS^+^ on different populations of CD11b^+^iNOS^+^ cells in the footpads of mice 6 weeks after infection with *L. major* Wt. * indicates statistical difference between groups of mice using Student’s *t* test (p < 0.05). Results are representative of two independent experiments. Three to 5 animals were used per group.

One of the mechanisms by which IFN-γ promotes resistance against *L.major* is by inducing iNOS expression in phagocytic cells (53, 56, 57). We evaluated the expression of iNOS at a keytime point (**Supplementary Figure 2C**), 6 weeks post-infection, when more parasites were first observed at the site of infection in GF mice (**Figure 1B**). At 6 weeks GF mice had the same frequency (**Figure 5A**, left panel) but lower numbers of iNOS^+^ myeloid cells (**Figure 5A**, right panel), along with reduced number and MFI of iNOS in non-inflammatory cells, monocyte-derived macrophage (Mon-Mϕ) and inflammatory monocytes (**Figure 5B**). Moreover, these cells harbored more parasites (**Figure 4**), likely due to their inability to be activated for efficient killing.

### Microbiota Imprints a Resistant Macrophage Activation Profile to *L. major* Infection

**Figure 6:**
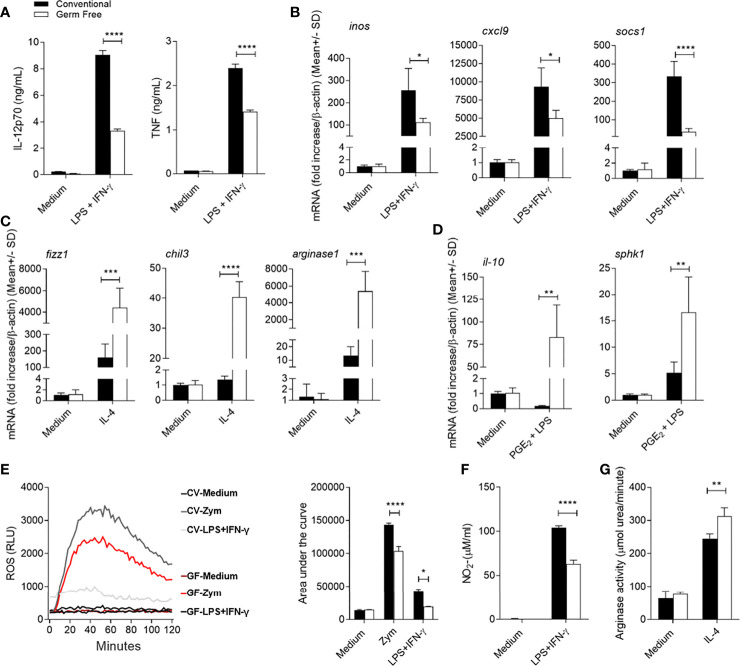
Influence of host microbiota on BMDM activation profile and response. **(A)** IL-12p70 and TNF in IL-12p70 and TNF assayed by ELISA in BMDM culture supernatant 48h and 24h after stimulation with LPS+IFN-γ, respectively. **(B)** Expression of *inos*, *cxcl9* and *socs1* mRNA in BMDM by real-time PCR after stimulation with LPS+IFN-γ for 24h. **(C)** Expression of *fizz1*, *chil3* and *arg1* mRNA in BMDM by real-time PCR after stimulation with IL-4 for 24h. **(D)** Expression of *Il10* and s*phk1* mRNA in BMDM by real-time PCR after stimulation with PGE_2_+LPS for 24h. **(E)** Production of reactive oxygen species (ROS) by BMDM unstimulated or stimulated with Zymosan (Zym); or with LPS + IFN-γ for 2h. **(F)** Nitrite production in the supernatant of BMDM by the Griess method, after 48h of stimulation with LPS + IFN-γ. **(G)** Measurement of arginase activity in BMDM after 48h of stimulation with IL-4. *, **, ***, **** indicates statistical difference between the two groups at the same time point using two-way ANOVA and post-Bonferroni test (p < 0.05; p < 0.01; p < 0.001; p < 0.0001). Results are representative of three independent experiments.

Our data indicates that the presence of the microbiota influences the activation phenotype of the host phagocytic cells during *L. major* infection. Thus, we further characterized the influence of the microbiota on bone marrow derived macrophages (BMDM) from GF and CV mice that were generated and stimulated *in vitro* with exogenous factors to induce polarization into the different activation states. When BMDM from CV mice were stimulated with LPS+IFN-γ, they showed an inflammatory phenotype or classical activation profile (4). By comparison, BMDM derived from GF mice showed an impaired ability to polarize this response. Absence of host microbiota resulted in diminished production of both IL-12p70 and TNF (**Figure 6A**) in response to LPS+IFN-γ. Moreover, GF-BMDM showed reduced mRNA expression of classical activation marker genes, *inos*, *cxcl9* and *socs1* (**Figure 6B**). To evaluate the alternative activation profile associated with tissue repair, we stimulated GF-BMDM and CV-BMDM with IL-4 (4). GF-BMDM presented higher expression levels of genes associated with wound healing, including *rentlα*, *chil3* and *arg1* (**Figure 6C**). When stimulated with PGE_2_+LPS, macrophages become activated into a suppressive phenotype (4), with GF-BMDM presenting higher expression of *il10* and *sphk1* (**Figure 6D**). Thus, GF-BMDM demonstrate an enhanced capacity to respond to activation signals leading to functions associated with tissue repair, resolution, and suppression of inflammation, whereas CV-BMDM are more readily driven to pro-inflammatory roles.

Next, we evaluated the effector response of BMDM from both groups, including production of microbicidal free radicals and their derivatives. GF-BMDM produced less ROS when stimulated with LPS+IFN-γ or with Zymosan, a classical stimulus for induction of ROS (**Figure 6E**). Consistent with their reduced level of iNOS mRNA expression (**Figure 6B**), GF-BMDM produced lower amounts of $NO_{2}^{-}$ (**Figure 6F**). By contrast, an increased arginase I activity (**Figure 6G**) in GF-BMDM after IL-4 stimulation was observed. These data suggest that microbiota drives macrophage polarization and function towards a resistant profile for *L. major* infection.

### Changes in the Microbiota Directly Influence *Leishmania major* Infection Development

**Figure 7:**
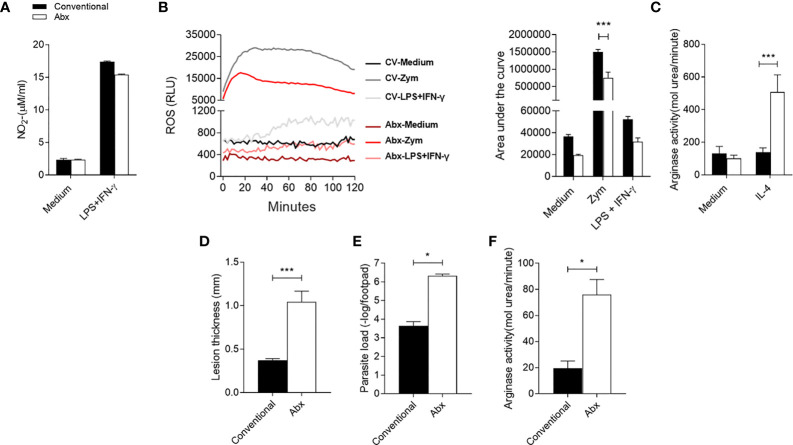
Dysbiosis alters *in-vitro* macrophage activation status and impairs control of infection *in vivo*. **(A)** Nitrite production in the supernatant of BMDM by the Griess method, after 48h of stimulation with LPS+IFN-γ. **(B)** Production of reactive oxygen species (ROS) by BMDM unstimulated or stimulated with Zymosan (Zym) or with LPS+IFN-γ for 2h. **(C)** Measurement of arginase activity in BMDM after 48h of stimulation with IL-4. **(D)** Lesion thickness in footpad 6 weeks post infection. **(E)** Quantification of parasite load by limiting dilution of the infected footpads 6 weeks post infection. **(F)** Arginase activity in footpad lysate 6 weeks after infection. *, *** indicates statistical difference between the two groups at the same time point using two-way ANOVA and post-Bonferroni test (p < 0.05; p < 0.001). Results are representative of three independent experiments. Three to 5 animals per group.

To gain more insight into the effect of the microbiota during *L. major* infection, deliberate manipulations of the mouse microbiota prior to BMDM assays and *in vivo* infection were performed. Microbiota of the CV mice were depleted with an antibiotic cocktail treatment once a week 2 weeks before harvest, and the effector response of BMDM from CV untreated and Abx-treated mice was evaluated. Our data indicate a global reduction of 99% in aerobic bacteria, gram-positive lactic acid bacteria – *Lactobacillus* and *Streptococcus* genera for example – and gram-negative Enterobacteriaceae (**Supplementary Figure 3A**). In addition, Abx-treated mice developed mega-cecum (**Supplementary Figure 3C**) as were observed in GF mice (**Supplementary Figure 3D**) - a remarkable characteristic of GF mice and great feature of microbiota depletion (58). No difference was observed in the production of $NO_{2}^{-}$ (**Figure 7A**) by BMDM when stimulated by LPS+IFN-γ. However, Abx-BMDM produced less ROS in response to Zymosan (**Figure 7B**). In addition, the Abx-BMDM presented higher arginase activity (**Figure 7C**) than CV-BMDM in the presence of IL-4. These data suggest that the effector response of macrophages was influenced by changes generated in the already established microbiota. In addition, they show that the influence of the microbiota on the activation of macrophages is a constant process and does not depend on early exposure during embryonic or neonatal development. Lastly, we treated CV animals with an antibiotic cocktail for 2 weeks prior to *L. major* infection and maintained treatment after infection until harvest. The animals in the Abx group presented larger lesions, higher parasite load (**Figures 7D–E**) and higher local arginase activity (**Figure 7C**) at the sixth week after infection, showing again the importance of the microbiota to the outcome of the disease.

**Figure 8:**
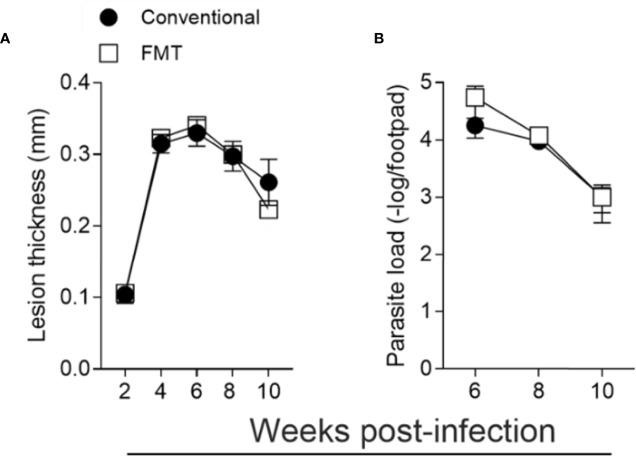
Microbiota is essential for the control of *L. major* infection. GF mice were conventionalized by fecal transfer and co-housing as described in *Methods*. **(A)** Lesion thickness in footpad was monitored every two weeks up to 10 weeks post infection. **(B)** Quantification of parasite load by limiting dilution 6 to 10 weeks post infection. The results are representative of two independent experiments. Four animals were used per group at each time point.

We also reconstituted the microbiota of GF mice by fecal microbiota transfer (FMT) (**Supplementary Figure 3B**). The reconstituted group was cohoused with CV animals for 2 weeks prior to *L. major* infection. We observed that reconstitution of the microbiota reverted the susceptibility phenotype presented by GF mice, with the course of infection and parasite loads indistinguishable between the FMT and CV animals (**Figures 8A, B**). Taken together, these data show that the microbiota are essential for the development of an effector response capable of controlling *L. major* infection.

## Discussion

In this study, we explored the *in vitro* activation phenotype of BMDMs under germ-free conditions. Employing flow cytometry, we characterized and compared the numbers and activation status of innate cell subsets in footpad lesions from Swiss germ-free mice, which are permissive to the infection by *L. major*, against conventional mice, which control this infection. In addition, by manipulating the presence of microbiota, either by FTM into germ-free mice, or by antibiotic treatment to deplete the gut microbiome in conventional mice prior to infection, we show the systemic impact of gut microbiota in activation of relevant phagocytic cells for the parasite.

Our data show that the absence of microbiota impacts the development of the disease by *L. major* infection. As previously reported, in the absence of microbiota higher numbers of parasites and larger lesions were observed (25). We confirm that no impairment of Th1 priming is associated with the higher susceptibility phenotype, as seen by the same levels of IL-12, IFN-γ, and TNF production by specific recall of *Leishmania* antigen response in the draining lymph nodes. Also, the levels of IL-4 and IL-10 were similar between the groups, thus, neither a polarized Th2 immunity or a stronger regulatory phenotype mediated by IL-10 is associated with the failure to contain parasite growth in the germ-free mice. Naik et al., using an intradermal model of infection with C57BL/6 mice, suggested that the skin microbiota exert dominant influence in skin immune response than gut microbiota and modulate T helper function through IL-1R1/MyD88 signaling pathway. Germ free mice were more permissive to *L. major* replication, presenting smaller lesions and less necrosis than specific-pathogen free mice. These outcomes were related to smaller numbers of IFN-γ and TNF-α T CD4 producing cells at the site of infection (37). In this work we have not addressed either the local *Leishmania* specific T cell response regarding to cytokine production or any T cell response towards skin microbiota that might occur during the dysbiosis promoted by the infection (59). We did find higher arginase I activity in lesions from germ-free mice, despite the same levels of IL-4 and IL-10 production by specific recall of *Leishmania* antigen response in the draining lymph nodes, which can induce arginase I expression (60), and are associated with parasite replication (23, 49). These data lead us to compare the innate and adaptive cell infiltrate between the two groups. We did not observed differences in neither frequency or numbers of B cells and CD4^+^ and CD8^+^ T cells at the site of infection or dLNs. Interestingly, at late stages of the infection, we found more dermal macrophages, a self-renewal population, probably due to higher local proliferation and more monocyte-derived macrophages in germ-free lesions. By tracking infected cells using RFP^+^ parasites, we observed more infected dermal macrophages and monocyte-derived macrophages in the GF lesions, as well as a higher RFP MFI in the infected inflammatory monocytes and monocyte-derived macrophages. Altogether, these data indicate that these cells might constitute a niche where the parasites are replicating, even in a context of Th1 immunity. Resident dermal macrophages represent a niche for *L. major* Seidman strain replication in the context of a highly polarized Th1 immune response, similar to the observations in our model. The dermal macrophages required IL-10, and localized delivery of IL-4 from eosinophils, in order to maintain their M2-like activation program (14, 24). Inflammatory monocytes and monocyte-derived macrophages represent a niche for parasite replication in other experimental models, such as infection by *L. amazonensis*, *L. donovani* and *L. major* (12, 13, 15, 17). Importantly, the control of infection by the self-healing *L. major* strain, which induces a strong Th1 immune response in C57BL/6 mice, still requires weeks after the onset of the adaptive immunity to be obtained (61, 62). Thus, the healing *versus* nonhealing phenotypes established after the infection by Fn or Seidman, respectively, L. *major* strains is only observed at later time points after infection (14, 63). Here we observed the same trend, where only after 6 weeks post-infection germ-free mice presented more parasites at the site of infection, which is also associated with the higher numbers of the host cells that might provide the niche for the parasite replication (dermal macrophages and monocyte-derived macrophages). It is possible that a certain threshold of innate phagocytic cells might have to get activated by IFN-γ, leading to iNOS expression and nitric oxide production, prior to the control of parasite load and lesion size in conventional mice (21). Nitric oxide is, so far, the main microbicidal molecule described as responsible for *L. major* killing by phagocytic cells (64, 65). We found an impairment of iNOS expression in those cell subsets in the absence of the microbiota at the site of infection, but still some phagocytic cells can respond to IFN- γ. Thus, some degree of parasite killing still happens in germ-free mice, which might explain, in part, the reason why the differences between GF *versus* CV parasite load only appear at late time point of the disease. Taken together, our data showed that, despite a Th1 environment, the lack of microbiota impacts the overall capacity of these phagocytic cells to respond to IFN-γ. On the other hand, as seen by higher Arg1 activity in these lesions, the absence of microbiota conditions these phagocytic cells to express a wound-healing and suppressive phenotype.

Here we used differentially stimulated BMDMs to acquire three different activation phenotypes: pro-inflammatory, wound-healing and suppressive, to evaluate how the microbiota would influence on these activation programs. It has already been shown that germ-free skin resident macrophages at steady state maintain their alternative activation phenotype (40). Our data show that in the absence of systemic microbiota modulation BMDMs from germ-free mice have an impaired capacity to polarize towards a pro-inflammatory phenotype and show a stronger polarization towards a wound-healing or suppressive phenotype. The inefficiency of macrophages to kill the parasite and why they function as permissive cells during late times of infection could be explained by the microbiota modulation of their cell activation status. In addition, peritoneum-derived macrophages from GF present reduced capacity for phagocytosis (42) and *L. major* killing (25). We showed that when activated by LPS+IFN-γ the *inos* mRNA is highly expressed in CV-BMDM, leading to higher production of the microbicidal molecules associated with killing of intracellular forms of *L. major* (64, 66). On the other hand, GF-BMDM activation, whether towards an alternative activation profile – driven by IL-4 and associated to wound healing and tissue repair – or a regulatory profile – marked by increased IL-10 production, conditions mice to susceptibility during *L. major* infection. IL-4 inhibits iNOS expression and consequently NO production (67, 68). GF-BMDM also show increased *arginase I* mRNA expression and protein activity, increasing the polyamines available in the intracellular compartment. These compounds are used by *L. major* for replication (69, 70) and this phenotype contribute for susceptibility to cutaneous leishmaniasis (71). The effect of IL-10 on macrophages leads to inhibition of their pro-inflammatory status, reducing IL-12, TNF and nitric oxide production (68, 72, 73). IL-10 is also involved in the susceptibility of *L. major* infection making macrophages refractory to IFN-γ activation (74). Thus, GF macrophages are refractory to classical activation stimuli. In a septic shock model, healthy host microbiota drives an inflammatory response towards increased TNF production and cell influx to the inflammatory site, whereas germ-free mice showed smaller cellular infiltration and TNF production. In this case, the presence of host microbiota was harmful to the organism, as these mice presented increased mortality. This phenomenon occurs due to increased levels of IL-10 at steady state and during inflammation in GF mice (75–77). In addition, gut dysbiosis, generated by antibiotic-treatment, is responsible for shifting macrophage polarization systemically. Alveolar lung macrophages after Abx-treatment polarize towards to alternatively activated profile, upregulating the expression of *arg1*, *chil33* and *rentlα* mRNA (78).

After antibiotic cocktail treatment, BMDM from conventional mice presented similar activation profile to GF-BMDM, indicating the constant modulation of host microbiota over the precursor cells in the bone marrow. In addition, the healthy microbiota is associated with good prognosis of cutaneous leishmaniasis disease as dysbiosis observed during infection is related to severity in humans and in mouse model (38). Microbiota derived compounds may start to shape the immune system before birth or the establishment of the microbiota in the organism by transfer of microbial products to the fetus through maternal antibodies and breastfeeding (26). It is important to mention that the antibiotic treatment was enough to induce susceptibility during *in vivo* infection even though *in vitro* activation of BMDMs by LPS+IFN-γ led to the same NO production comparing to the control group. We can speculate that LPS+IFN-γ might induce a stronger activation in BMDMs *in vitro* than what is observed during the *in vivo* infection by *L. major*. Thus, in this context, phagocytic cells in Abx-treated mice could still have an impairment response to the classical activation. Similar results have been observed during *Listeria monocytogenes* infection. Systemic infection with *L. monocytogenes* in germ-free and oral-antibiotic-treated mice display higher pathogen burden and mice death in comparison with control mice (30, 79). Moreover, the monoassociation with a probiotic *Lactobacillus* (35) or recolonization of germ-free mice with a complex microbiota rescue the mice from infection (30). Monoassociation of germ-free mice is enough to rescue serum levels of nitrate and production by peritoneal derived cells (35) indicating the remaining bacteria after antibiotic treatment could sustain nitric oxide production in Abx-BMDM. In our model, the fecal microbiota transfer and the antibiotic cocktail treatment reinforce the role of microbiota on disease outcome. Rescuing the host microbiota by transfer of feces from healthy mice recovers the ability to control the infection. On the other hand, dysbiosis generated by antibiotic cocktail treatment impaired the ability to control the disease. Corroborating the idea of systemic influence of microbiota in immune cell development (27, 28), our study adds new evidence to support a role for the host microbiota in systemic cell activation and precursors development.

Although the bone marrow is not normally colonized by microorganisms, microbial metabolites and microbial-associated molecular patterns (MAMPs) can still impact the physiology of this organ. For instance, steady state hematopoiesis depends upon microbiota complexity, mediated by toll-like receptors (TLRs) signaling, where the numbers of mature granulocytes in conventional mice are higher compared to germ-free or antibiotic-treated mice (29, 80, 81). The microbiota also influences monocyte emigration from bone marrow and monocyte derived-macrophage differentiation and activation in several different tissues (27, 32, 40, 82, 83). Microbial metabolites also interfere with cell production, including macrophages and neutrophils, in the bone marrow (29, 82). We propose that the macrophage activation modulation mediated by microbiota might happens trough either/both recognition of MAMPs or microbial metabolites by dermal macrophages at the skin or mononuclear cell precursors at the bone marrow. TLRs are a family of widely expressed pattern-recognition receptors that detect microbial products, such as LPS and LTA, recognized by TLR4 and TLR2 respectively (84). Interestingly, the treatment of germ-free mice with LPS, LTA or CpG (TLR9 agonist) is enough to transiently switch the systemic immune response from anti-inflammatory into a pro-inflammatory response during a model of intestinal ischemia/reperfusion injury and induce protection against pulmonary infection by *Klebsiella pneumoniae* (85). While still controversial, the activation of TLRs during *Leishmania* infection has also been associated with a protective phenotype. TLR4 contributes to efficient control of *L. major* infection acting by controlling parasite replication (86, 87). Another mechanism involving TLR4 is its activation by neutrophil elastase eliciting microbicidal activity responsible for *L. major* killing by macrophages (88). In human cutaneous leishmaniasis the up-regulation of TLR2/TLR4 expression in monocytes correlates with disease outcome (89), their up-regulation expression by macrophages and monocytes is related to healing lesions (90) by increasing ROS and NO production (91). Disruption of MyD88, the universal adapter for TLRs, excluding TLR3, block the proper Th1 immune response against *L. major* (92, 93). Other study using a transgenic mouse deficient for endosomal nucleic acid sensing TLRs (TLR3, TLR7 and TLR9) showed increased parasitism and bigger lesion after 6 weeks post infection. Susceptibility is associated to highly levels of IL-10, reduced IFN-γ and IL-12 production (94). Therefore, proper microbiota-derived signaling is determinant for leishmaniasis infection.

Other relevant commensal feature is bacterial metabolites that gained more insight in the last decade due to the influence in host homeostasis and immune response (95). Short chain fatty-acids (SCFAs), such as acetate and butyrate, and lactate are metabolic products of gut commensals that get into the bloodstream and are found on peripheral circulation (96). They can act as inhibitors of histone deacetylases (HDACs) and can promote the accumulation of transcriptionally permissive acetyl modifications at gene enhancers and promoters, thus acting as modifiers of epigenetic responses (97, 98). Epigenetic modifications regulate the differentiation of all innate cells from common myeloid progenitors and are responsible for the distinct phenotypic variations present within differentiated cells, such as macrophage polarization status by HDAC (99). Activation of PRRs signaling is also epigenetically regulated (100), indicating a fine tuning and regulation of immune response dependent of microbial sensing. Thus far, there is no evidence of microbiota derived metabolites influence during *Leishmania sp* infection. Nevertheless, the presence of host microbiota – consequently presence of SCFAs and lactate - promote efficient antiviral immunity. Epigenetic alteration in macrophages and dendritic cells that promotes IFN type I inflammatory response against viral infections occurs in conventional mice but not in germfree mice (101). One of the dominant microbial metabolites, butyrate, is strongly reduced in mice after antibiotic treatment, which is also seen in germ-free mice (102, 103). Presence of butyrate inhibits HDAC3 function in macrophages promoting the polarization towards pro-inflammatory activation. Butyrate stimulate microbicidal activity in macrophages as they produce high levels ROS and present enrichment transcription profile for killing intracellular pathogens and defense response to bacteria (104). Thus, decreased availability of butyrate in GF and Abx groups could lead to an inappropriate antimicrobial program in macrophages resulting in the impaired response against *L. major* by those cells.

Here we have shown that the modulation of monocyte and macrophage activation status by the host microbiota is essential for proper control of *L. major* infection in the skin. We described refractory inflammatory response and diminished production of effector free radicals in macrophages from GF mice. By contrast, macrophages derived from CV mice display a significantly greater capacity of responding to pro-inflammatory stimuli. This dichotomy is particularly striking by the increased NO production by CV-BMDM and increased arginase activity in GF-BMDM. In addition, we saw reduced amounts of dermal macrophages and monocyte-derived macrophages able to kill the parasite *in vivo*. Consequently, it seems clear that microbiota priming of immune cells is essential to proper responses against *L. major*, and likely against other intracellular pathogens.

## Author’s Note

CAPES is currently cutting funds to graduate programs, even those of recognized excellency. CNPq has not launched any calls for basic research proposals for the last two years and is also cutting fund for scholarships. Brazil is currently dismantling research groups and facilities due to lack of funding. The state agency, FAPEMIG, which previously funded the authors in Brazil, owes the authors grant money that has been approved and not paid. This paper was written under great distress due to the coronavirus pandemic and threats against Democracy and Science in Brazil.

## Data Availability Statement

The datasets presented in this study can be found in online repositories. The names of the repository/repositories and accession number(s) can be found below: http://hdl.handle.net/1843/34453 dissertation and thesis repository of the Universidade Federal de Minas Gerais. This deposit is compulsory to all thesis and dissertations defended by recognized graduate programs in Brazil.

## Ethics Statement

The animal study was reviewed and approved by Comissão ética de utilização animal, Universidade Federal de Minas Gerais, protocols number 110/2012 and 266/2017.

## Author Contributions

ML conceptualized the project, designed, and performed the experiments, analyzed, and interpreted data, and wrote the manuscript. LS designed, performed the experiments, interpreted data, and wrote the manuscript. DS designed and supervised the experiments, wrote the manuscript. MC and LV conceptualized the project, designed, and supervised the experiments, analyzed, and interpreted data, and wrote the manuscript. All authors contributed to the article and approved the submitted version.

## Funding

ML is CNPq fellow. LS is a CAPES fellow. MC is a CNPq fellow. LV is a CNPq fellow. This study was financed in part by Coordenação de Aperfeiçoamento de Pessoal de Nível Superior, Brazil (CAPES) Finance Code 001, and CNPq grants number 304588/2013-0, 309789/2017-5 and 400729/2014-8, and FAPEMIG grant number CBB APQ-01993-12. This work was funded in part by the intramural research program of the NIAID.

## Conflict of Interest

The authors declare that the research was conducted in the absence of any commercial or financial relationships that could be construed as a potential conflict of interest.

## Publisher’s Note

All claims expressed in this article are solely those of the authors and do not necessarily represent those of their affiliated organizations, or those of the publisher, the editors and the reviewers. Any product that may be evaluated in this article, or claim that may be made by its manufacturer, is not guaranteed or endorsed by the publisher.
