# Supplementary material for: Resistance Against Leishmania major Infection Depends on Microbiota-Guided Macrophage Activation
Source: Front Immunol. 2021 Oct 20;12:730437. doi: 10.3389/fimmu.2021.730437 (PMC8564857; doi:10.3389/fimmu.2021.730437)
Supplement: Supplementary file 1 [file DataSheet_1.docx]

Supplementary Material

# Supplementary Table

**Supplementary Table 1.** **Primers sequence**

| **Gene** | **Forward** | **Reverse** |
| --- | --- | --- |
| *inos* | 5’-CCCTTCCGAAGTTTCTGGCAGCAGC-3’ | 5’-GGCTGTCAGAGCCTCGTGGCTTTGG-3’ |
| *cxcl9* | 5’-GGGATTTGTAGTGGATCGTGC-3’ | 5’-GGAGTTCGAGGAACCCTAGTG-3’ |
| *actinb* | 5’-AGGTGTGCACCTTTTATTGGTCTCAA-3’ | 5’-TGTATGAAGGTTTGGTCTCCCT-3’ |
| *arginase I* | 5’-CTGGCAGTTGGAAGCATCTCT-3’ | 5’-CTGGCAGTTGGAAGCATCTCT-3’ |
| *ym1* | 5’-AGAAGGGAGTTCAAACCTGGT-3’ | 5’-GTCTTGCTCATGTGTGTAAGTGA-3’ |
| *relmα* | 5’-AATCCAGCTAACTATCCCTCCA -3’ | 5’-CAGTAGCAGRCATCCCAGCA-3’ |
| *sphk1* | 5’-ACAGCAGTGTGCAGTTGATGA-3’ | 5’-GGCAGTCATGTCCGGTGATG-3’ |
| *il10* | 5’-CCAGTTTTACCTGGTAGAAGTGATG-3’ | 5’-TGTCTAGGTCCTGGAGTCCAGCAGACTCAA-3’ |
| *socs1* | 5’-ACACTCACTTCCGCACCTTC-3’ | 5’-GAAGCCATCTCCACGCTG-3’ |

**2 Supplementary Figures**

#
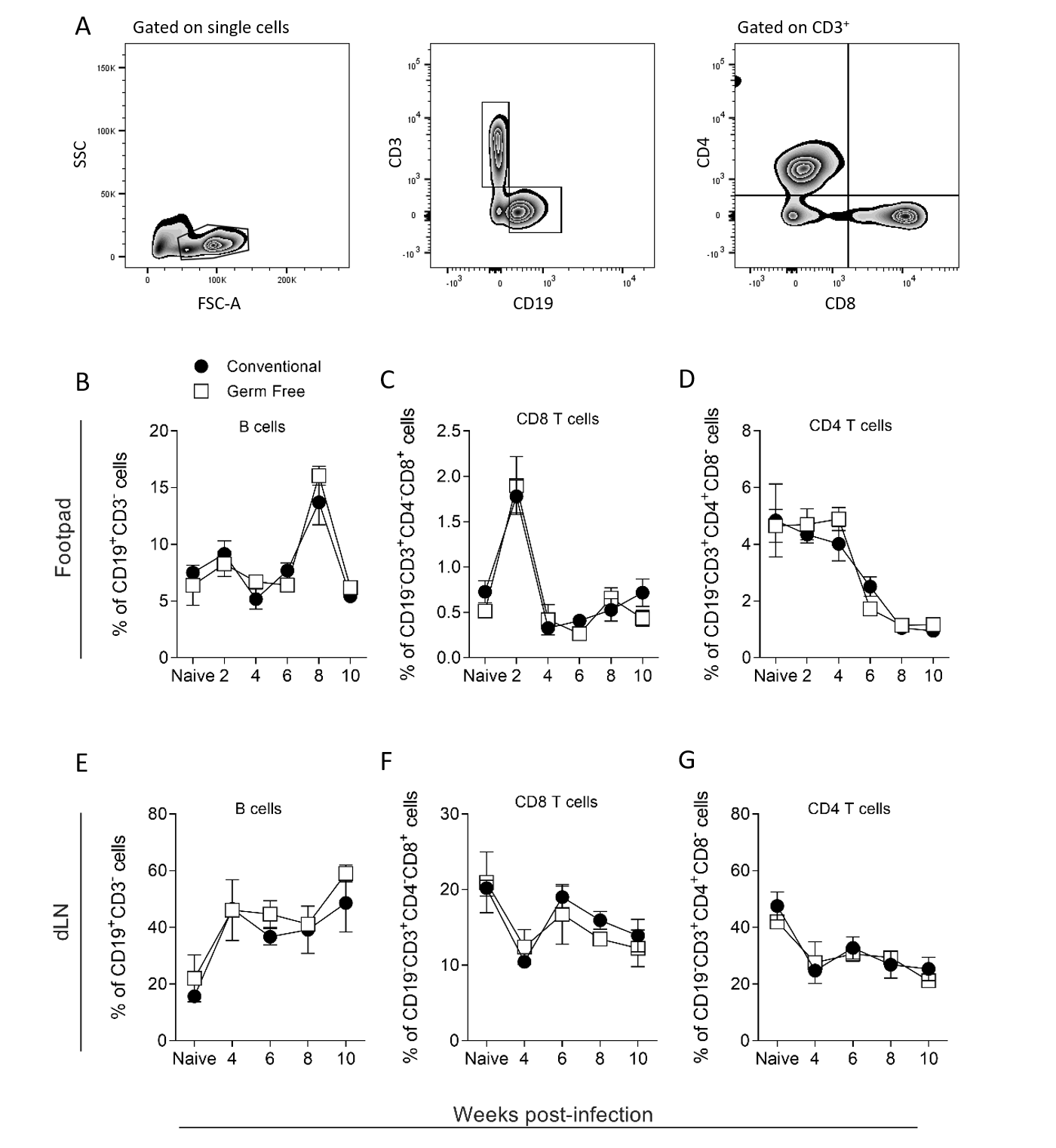


**Supplementary Figure 1.** **Host microbiota did not alter the proportion of infiltrating lymphocyte subsets.** (A) Gating strategy used for analysis of lymphoid cell populations in the footpads and in the draining lymph nodes of uninfected and *L. major* infected mice*.* (B-D) Percentage of lymphocytes in the footpads of uninfected animals and infected from the second to the tenth week after infection. (E-G) Percentage of lymphocytes in the popliteal lymph nodes of uninfected and 4-10 weeks infected mice. The results are representative of two independent experiments. Four to 6 animals per group were used per time.

**
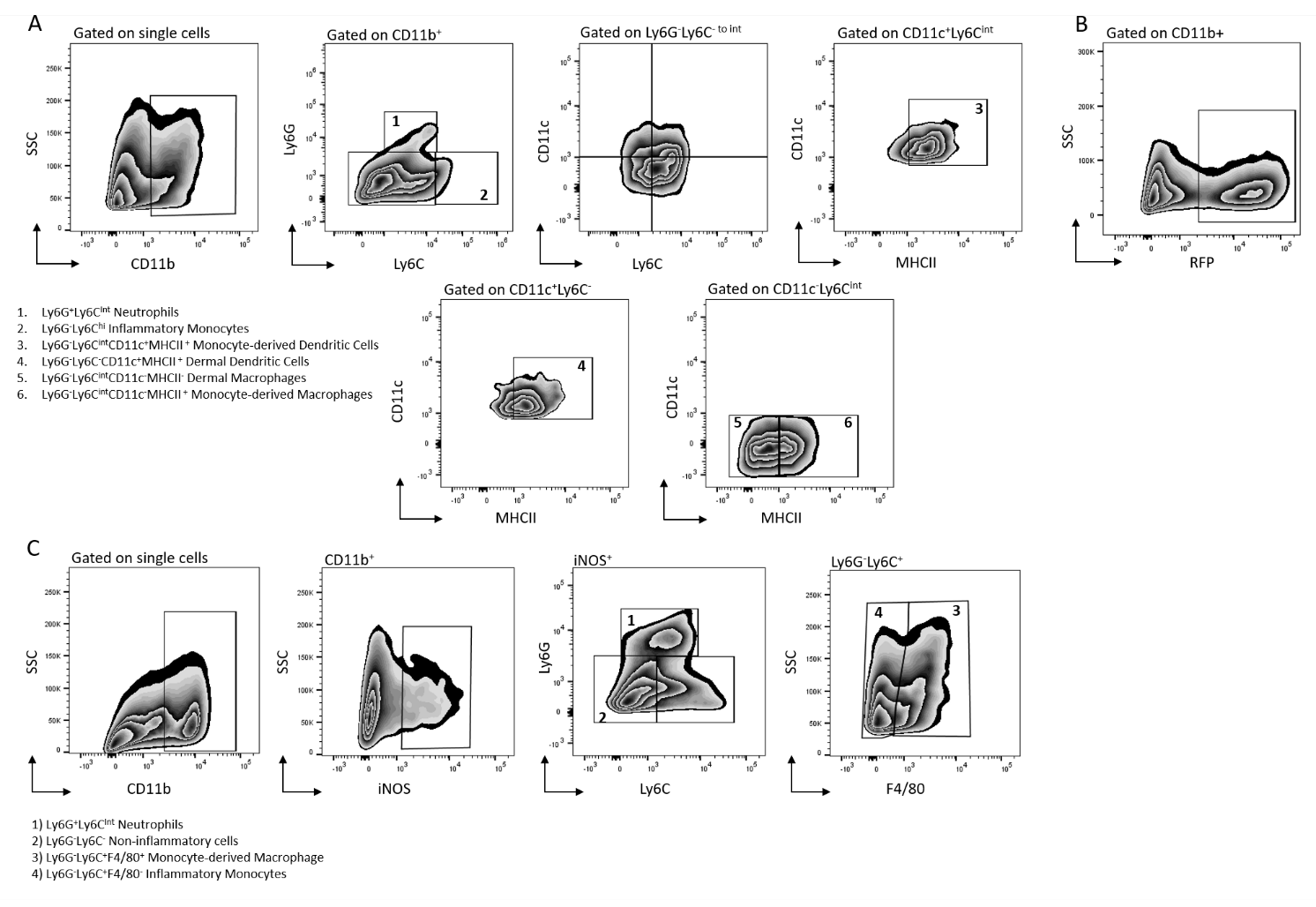
Supplementary Figure 2.** **Gating strategy for cellular composition during infection.** (A) Gating strategy used for analysis of myeloid cell populations in the footpads of uninfected and *L. major* infected mice. (B) Gating strategy for CD11b^+^RFP^+^ cells used for analysis of infected myeloid cells subsets in the footpads of mice infected with *L. major* RFP. (C) Gating strategy used for analysis of iNOS^+^ myeloid cell populations in the footpads 6 weeks after infection with *L. major*.

**
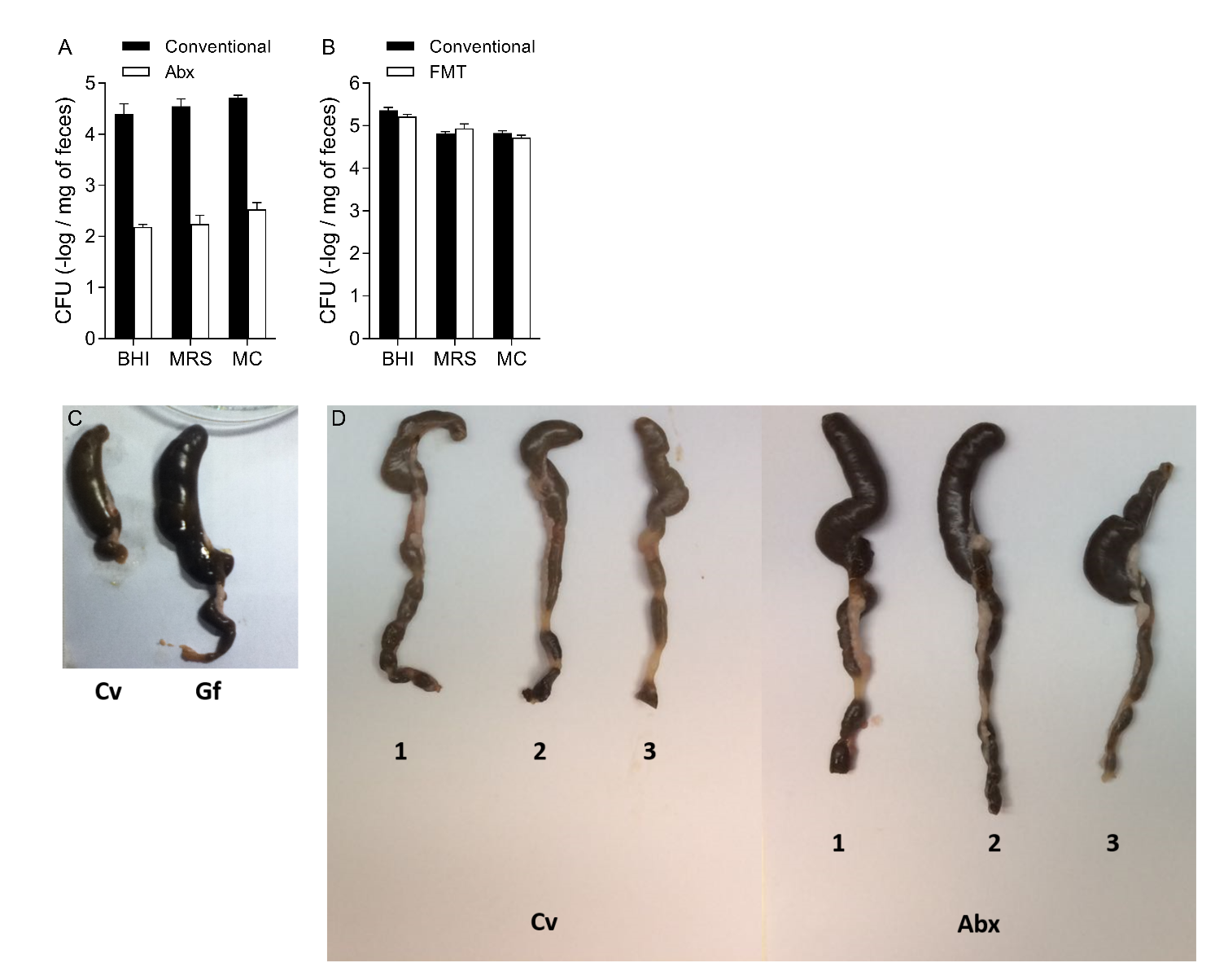
**

**Supplementary Figure 3. Control of** **host microbiota modulation.** (A-B) Control of host microbiota depletion using antibiotics cocktail (A) and reconstitution by fecal microbiota transplant (B) using colony forming units (CFU) were performed after completion of each protocol of microbiota modulation and prior to the infection and harvest. BHI = brain heart infusion agar; MRS = De Man, Rogosa and Shaper agar; MC= MacConkey agar **(**C-D) Representative images of cecum from conventional (Cv) and mega-cecum of germ-free (Gf) and antibiotic (Abx) treated mice before harvest and infection.
